# *ER24/1 !*: The greatest emergency of our time

**DOI:** 10.1007/s00068-023-02314-9

**Published:** 2023-06-27

**Authors:** Elaine Pei-Jing Xiao-Wei van Ee, Nicolas D. John Barker, John Howard Barker

**Affiliations:** 1 Leiden University Medical Center, Leiden, The Netherlands; 2 TU Delft, Delft, The Netherlands; 3 JW Goethe University, Frankfurt am Main, Germany

The artistic video presentation accompanying this editorial, titled “*ER24/1 !”* was created by the Dutch artist, Maria Koijck, and is the fourth in a series of five different and unique artistic creations, each assembled using medical waste collected from procedures in five different subspecialties [1, 2]. The medical waste represented here in *ER24/1 !* consists of 146 kg (139 bags) of single-use, disposable waste generated while treating 92 patients, during 24 h on a weekday at a single Emergency Department in the Netherlands. Once collected, the discarded waste was assembled and captured on film by the artist, and edited by Eva Glasbeek. The accompanying music was composed and performed by Lucas Raimbault.

*Background of “ER24/1 !”:* Maria Koijck [3] was diagnosed with breast cancer in 2009 and treated with a mastectomy and breast reconstruction at the University Medical Center Groningen (UMCG), in the Netherlands. Following this life-altering experience, she chose—through her art—to turn this traumatic experience into something positive. Shocked by the amount of discarded single-use medical supplies used in her breast reconstruction, she asked the surgical staff to collect the pre-, intra-, and post-operative waste, and with it, she created her first work of art using medical waste [4]. She hoped that her creation would make others aware of the enormous amount of waste generated from a single surgery—her own surgery. In the artist’s own words, these creations are “to raise awareness in the medical community of the shocking amount of waste used in these medical procedures. Hopefully, by highlighting this astonishing amount of waste, my art will open people’s eyes and motivate them to act to help reduce medical waste” [1].

The World Health Organization (WHO) defines healthcare systems as “*organizations, people and actions whose primary intent is to promote, restore or maintain health*” [5]. Contrary to this definition, healthcare systems, through their disproportionally large environmental footprint, have an unintended negative impact on human health, and instead of promoting it, they cause significant disease, suffering, and death. Healthcare’s oversized environmental footprint is caused primarily by air pollution, through excessive energy use—electricity, gas, steam, air conditioning, and incineration of waste [6]. Air pollutants, in the form of particulate matter, carbon monoxide, ozone, nitrogen dioxide, and sulfur dioxide increase the incidence and severity of illnesses like stroke, heart disease, lung cancer, and acute and chronic respiratory disease [7–9]. To put this into perspective, “Air pollution exceeds malaria in the number of premature deaths globally by a factor of 19; it exceeds violence by a factor of 16, HIV/AIDS by a factor of 9, alcohol by a factor of 45, and drug abuse by a factor of 60." [10]. The Lancet Commission on Pollution and Health reports that “…pollution is responsible for approximately 9 million deaths per year, or 1 in 6 deaths worldwide” [11]. Healthcare systems cause up to 5% of global greenhouse gas emissions, equivalent to the annual emissions from 514 coal-fired power plants [12]. If the healthcare sector were a country, it would be the fifth largest greenhouse gas emitter on the planet. There is a strong correlation between the size of a country’s climate footprint and its health sector spending. Generally, the greater the spending, as measured by a country’s GDP, the higher the per capita healthcare emissions. Accordingly, the United States’ healthcare sector is the world’s number one greenhouse gas emitter, in both absolute and per capita terms, producing 7 times more emissions per person than China, and 57 times more per person than India. Of the air pollution attributable to the health sector, 71% is derived from the healthcare supply chain, through the production, transport, and disposal of goods such as pharmaceuticals and other chemicals, food and agricultural products, hospital equipment, single-use instruments, and excess packaging [12, 13].

Eighty-five percent of this waste is general, non-hazardous, and comparable to domestic waste, while the remaining 15% is hazardous material that may be infectious, chemical, or radioactive. Whether hospital waste is labeled as ‘hazardous’ or ‘non-hazardous’ influences how it is processed and disposed of. If non-hazardous waste is incorrectly labeled as ‘hazardous’, it is processed and disposed of using methods that cause significantly more emissions of pollutants and a greater environmental footprint. Every year, an estimated 16 billion injections are administered worldwide; however, not all the needles and syringes are correctly disposed of. Hospitals in the US generate over 29 pounds of waste per bed per day, or more than 5 million tons of waste each year [6]. A large portion of this waste is in the form of disposable, single-use plastic, a great deal of which could be reduced or eliminated [14].

Emergency departments (ED) make up a big part of the activity in a hospital and consequently are also major contributors to hospital waste production. Emergency departments in the United States attend approximately 140 million patients yearly [15]. Due to this large patient turnover and the high level of stress in busy emergency departments, those who work in these environments often do not prioritize sustainability and proper waste separation and/or disposal [16]. The 139 bags of medical waste represented here in *ER24/1 !* were generated treating 92 patients on 1 day, in 1 ED in the Netherlands. Imagine how much waste is generated treating the 6100 patients seen each day in the Netherlands [17], or the amount of waste generated in the 734,000 daily ED visits in Europe plus the US [15, 18]!

In 2019, a study describing a program to reduce ED waste in a regional hospital in Australia found that efforts to improve waste segregation and recycling failed due to poor compliance. The authors reported that “Staff felt that the process was time consuming and complicated and environmental services staff were seen mixing different waste bins together to simplify the process” [19]. In an urban tertiary-care academic medical center in the United States, researchers measured the amount of waste generated in their ED, over a period of 24 h (weekday). They reported 671.8 kg of total waste of which the majority (65%) was plastic, there were 200 unused items, and only 14.9% of the waste disposed of in red bags met the criteria for “hazardous” medical waste [20]. Several authors have proposed measures to improve routine waste management practices in EDs, including stricter recycling and sorting practices, restocking and donating unnecessary items, composting, diverting, and reducing food waste, electronic waste sorting, reusing items, and reducing low-yield laboratory testing [20–24]. Emergency departments interact with many other specialties and are, thus, uniquely positioned to lead hospital efforts and set an example of best practices for promoting sustainability measures. From a global perspective, the main barriers standing in the way of implementing sustainable practices in the healthcare sector include lack of awareness about the health hazards related to healthcare waste, inadequate training in proper waste management, absence of waste management and disposal systems, insufficient financial and human resources, and the low priority given to the topic [6].

While artists usually do not offer solutions to problems, their creations, in the form of literary, performing or visual art, can heighten our awareness leaving us with impressions that cause us to reflect, debate, and act. It is our hope that disseminating this artistic presentation and accompanying editorial throughout the medical community will help to chip away at these barriers by *heightening awareness* of the shocking amount of waste being generated and the resulting associated health hazards. We hope that the images featured and described in this presentation will cause our fellow healthcare colleagues to act, and while performing their busy daily routines, decide not to use that overpackaged item, or to clean and reuse or recycle that perfectly functional instrument, or not needlessly open that sterile instruments set, or dispose of non-hazardous and/or hazardous waste in the correct bin. These small changes in our busy daily routines, if performed by many, can make a big difference. Given that the healthcare community’s primary objective is to promote, restore, and maintain human health, to do so we must extend that care to our planet. After all, our patient’s health is inextricably tied to the health of our planet. We hope that this artistic presentation inspires our fellow healthcare colleagues to take action in caring for our planet’s health, with the same compassion and dedication they apply in their daily jobs caring for their patients.

### Supplementary Information

Below is the link to the electronic supplementary material.

Supplementary file1 (MP4 132069 kb)

Supplementary file2 (PNG 21256 kb)
