# Supplementary figures and images for: ER24/1 !: The greatest emergency of our time
Source: Eur J Trauma Emerg Surg. 2023 Jun 27;49(6):2323–5. doi: 10.1007/s00068-023-02314-9 (PMC10728227; doi:10.1007/s00068-023-02314-9)

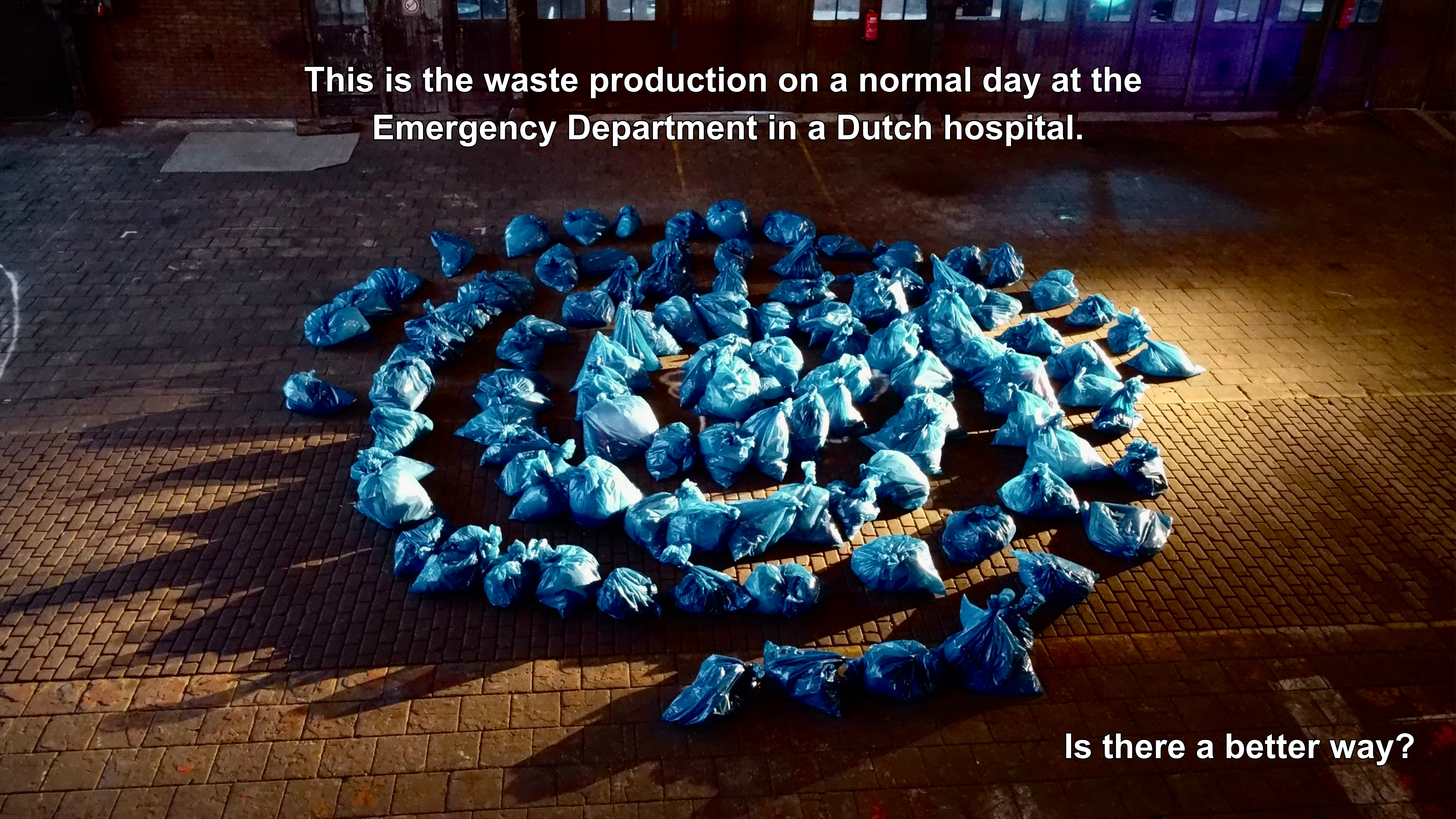

Supplement: Supplementary file 2 — Supplementary file2 (PNG 21256 kb) [file 68_2023_2314_MOESM2_ESM.png]
